# APOBEC3G targets human T-cell leukemia virus type 1

**DOI:** 10.1186/1742-4690-2-32

**Published:** 2005-05-19

**Authors:** Amane Sasada, Akifumi Takaori-Kondo, Kotaro Shirakawa, Masayuki Kobayashi, Aierkin Abudu, Masakatsu Hishizawa, Kazunori Imada, Yuetsu Tanaka, Takashi Uchiyama

**Affiliations:** 1 Department of Hematology and Oncology, Graduate School of Medicine, Kyoto University, 54 Shogoin-Kawaracho, Sakyo-ku, Kyoto 606-8507, Japan; 2 Department of Immunology, Graduate School and Faculty of Medicine, University of the Ryukyus, Uehara 207, Nishihara-cho, Nakagami-gun, Okinawa 903-0215, Japan

## Abstract

**Background:**

Apolipoprotein B mRNA-editing enzyme-catalytic polypeptide-like 3G (APOBEC3G) is a host cellular protein with a broad antiviral activity. It inhibits infectivitiy of a wide variety of retroviruses by deaminating deoxycytidine (dC) into deoxyuridine (dU) in newly synthesized minus strand DNA, resulting in G-to-A hypermutation of the viral plus strand DNA. To clarify the mechanism of its function, we have examined the antiviral activity of APOBEC3G on human T-cell leukemia virus type 1 (HTLV-1), the first identified human retrovirus.

**Results:**

In this study, we have demonstrated that overexpressed as well as endogenous APOBEC3G were incorporated into HTLV-1 virions and that APOBEC3G inhibited the infection of HTLV-1. Interestingly, several inactive mutants of APOBEC3G also inhibited HTLV-1 and no G-to-A hypermutation was induced by APOBEC3G in HTLV-1 genome. Furthermore, we introduced the human immunodeficiency virus type 1 (HIV-1) vif gene into HTLV-1 producing cell line, MT-2, to antagonize APOBEC3G by reducing its intracellular expression and virion incorporation, which resulted in upregulation of the infectivity of produced viruses.

**Conclusion:**

APOBEC3G is incorporated into HTLV-1 virions and inhibits the infection of HTLV-1 without exerting its cytidine deaminase activity. These results suggest that APOBEC3G might act on HTLV-1 through different mechanisms from that on HIV-1 and contribute to the unique features of HTLV-1 infection and transmission.

## Background

APOBEC3G, also known as CEM15 [1], is a host cellular protein which has a broad antiviral activity on a wide variety of retroviruses including HIV-1, other lentiviruses, and murine leukemia virus (MLV) [2-4]. The protein belongs to the Apobec superfamily of cytidine deaminases [5] and inhibits the infectivity of these viruses by being packaged into virions. During reverse transcription, it deaminates deoxycytidine (dC) into deoxyuridine (dU) in newly synthesized minus strand DNA, resulting in either G-to-A hypermutation of the viral plus strand DNA or degradation of dU-rich reverse transcripts [3,6-8], though several resent studies suggest cytidine deaminase adtivity is essential but not a sole determinant for antiviral activity of APOBEC3G. [7]. Most lentiviruses express an accessory protein called virion infectivity factor (Vif) which blocks the antiviral function of APOBEC3G by preventing its packaging into virions. Vif binds to APOBEC3G and induces its ubiquitination and subsequent degradation by the proteasome [9-13]. It has also been reported that APOBEC3G inhibits the replication of hepatitis B virus (HBV) without inducing G-to-A hypermutation [14]. This suggests that APOBEC3G has a broad antiviral activity not only on retroviruses but also on other viruses through different mechanisms from that on retroviruses.

HTLV-1 is a member of retroviruses which is the etiologic agent of adult T-cell leukemia(ATL) [15] and HTLV-1 associated myelopathy/tropical spastic paraparesis (HAM/TSP) [16]. HTLV-1 has a unique feature of its infectivity and transmission, that is, cell-to-cell contacts are necessary for HTLV-1 transmission, because HTLV-1-infected lymphocytes produce very few cell-free virions, of which, only 1 in 10^5^to 10^6 ^is infectious [17]. The fact that infusion of fresh frozen plasma from the seropositive individuals did not cause the transmission also supports the notion that living infected cells are essential for the transmission *in vivo *[18,19]. Furthermore, the genetic diversity of HTLV-1 is much lower than that of other retroviruses such as HIV-1, although the most frequent mutations in HTLV-1 are also G-to-A transitions [20]. In addition to *gag*, *pol*, and *env *genes, HTLV-1 genome has four open reading frame (ORF) regions at its 3' end, which encode regulatory proteins including Rex and Tax. Although the functions of other encoded proteins such as p12, p13, and p30 have been under investigation [21,22], any counterparts of HIV-1 Vif have not been identified in HTLV-1. These findings suggest the involvement of APOBEC3G in the characteristic infectious and genetic features of HTLV-1 and lead us to investigate this possibility.

In this report, we have investigated the antiviral activity of APOBEC3G on HTLV-1. We examined the packaging of APOBEC3G into HTLV-1 virions, induction of mutations in the viral genome, and regulation of the viral infectivity. Our finding would be a clue to understand the unique infectious mechanism of HTLV-1.

## Results

### APOBEC3G was incorporated into HTLV-1 virions

**Figure 1:**
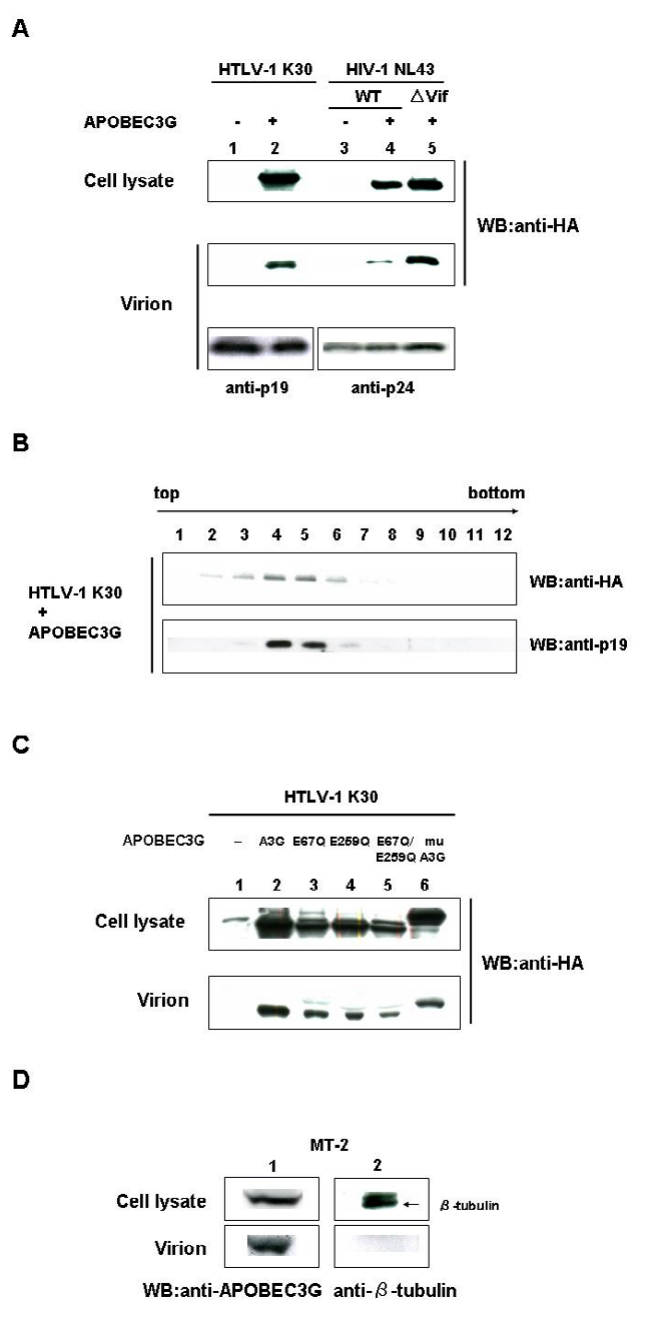
**Incorporation of APOBEC3G into HTLV-1 virions**. **(A) Overexpressed APOBEC3G was incorporated into HTLV-1 virions. **HEK293T cells were cotransfected with K30, pNL43-Luc (WT), or pNL43/Δvif-Luc (ΔVif) with or without an expression vector for HA-APOBEC3G. Western blotting was performed to detect HA-APOBEC3G in HEK293T cells and produced virions with anti-HA mAb. APOBEC3G was expressed in producer cells and efficiently incorporated into produced virions (lane 2). Expression of APOBEC3G and its incorporation into HIV-1 virions were reduced by expression of Vif as described previously (lane 4). Western blotting with anti-p19 and anti-p24 mAbs showed that similar amounts of virions were produced from each transfection (bottom panel). **(B) Incorporation of APOBEC3G was confirmed in HTLV-1 virions purified by sucrose density equilibrium gradient analysis. **HTLV-1 K30 virions were purified by sucrose density equilibrium gradient analysis. Gradient fractions were collected and used for analyzing incorporation of APOBEC3G into virions. APOBEC3G were detected and colocalized with HTLV-1 Gag (p19) proteins (lanes 4, 5). **(C) APOBEC3G, its mutants, and muAPOBEC3G were incorporated into HTLV-1 virions. **Expression vectors for HA-APOBEC3G, its mutants, or HA-muAPOBEC3G were cotransfected with K30 into HEK293T cells and APOBEC3G was detected with anti-HA mAb. HA-APOBEC3G, its mutants, and HA-muAPOBEC3G were all incorporated into virions. A3G and muA3G indicate human and murine APOBEC3G, respectively. E67Q, E259Q, and E67Q/E259Q were inactive mutants of human APOBEC3G that have a point mutation in N-terminal active site, C-terminal active site, and both, respectively, as described previously [7]. **(D) Endogenous APOBEC3G was also incorporated into HTLV-1 virions. **Western blotting with anti-APOBEC3G Ab revealed expression of endogenous APOBEC3G in MT-2 cells (lane 1, upper panel) and its incorporation into produced virions (lane 1, lower panel). No cytoplasmic proteins were detected with anti-β-tubulin mAb in MT-2 virions (lane 2, lower panel).

We first examined the incorporation of APOBEC3G into HTLV-1 virions. We transfected HEK293T cells with an infectious molecular clone of HTLV-1 (K30) and infectious molecular clones of HIV-1 with or without vif (pNL43-Luc or pNL43/Δvif-Luc, respectively) with or without an expression vector for HA-APOBEC3G and performed Western blotting to detect APOBEC3G in producer cells and produced virions. Incorporation of APOBEC3G was clearly detected in HTLV-1 virions produced from cells cotransfected with HTLV-1 K30 and APOBEC3G expression vector (Fig. 1A, lane 2). Expression of APOBEC3G and its incorporation into HIV-1 were reduced by expression of Vif as reported previously (Fig. 1A, lane 4) [3,4,7,8]. Packaging of APOBEC3G into virions was also confirmed by Western blotting of HTLV-1 K30 virions purified by sucrose density equilibrium gradients method (Fig. 1B). APOBEC3G were detected and colocalized with HTLV-1 Gag (p19) proteins (lanes 4, 5), indicating the incorporation of APOBEC3G into HTLV-1 virion. APOBEC3G mutants and murine APOBEC3G (muAPOBEC3G) were also detected in HTLV-1 virions (Fig. 1C). Since we detected the incorporation of overexpressed APOBEC3G into HTLV-1 virions, we next examined the incorporation of endogenous APOBEC3G into HTLV-1 virions using an HTLV-1 producing cell line, MT-2, which expressed endogenous APOBEC3G (Fig. 1D, lane 1, upper panel). We also detected the incorporation of endogenous APOBEC3G in HTLV-1 virions produced from MT-2 cells (Fig. 1D, lane 1, lower panel). An abundant cytoplasmic protein, β-tubulin, was not detected in MT-2 virion, which excluded the possibility of contamination of the MT-2 virion preparations by cytoplasmic proteins (Fig. 1D lane 2). These indicate that APOBEC3G cannot be excluded from HTLV-1 virions.

### HTLV-1 infectivity was inhibited by APOBEC3G

**Figure 2:**
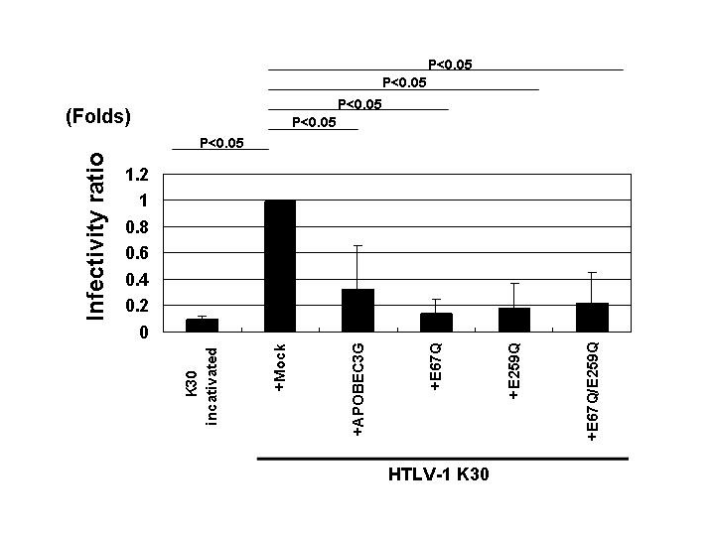
**Inhibition of HTLV-1 infection by APOBEC3G**. APOBEC3G as well as its mutants inhibited the infectivity of HTLV-1. Infectivity of HTLV-1 was measured as described in Materials and Methods. HTLV-1 proviral DNA load in target SupT1 cells was suppressed by APOBEC3G and its mutants to the level of that of heat-inactivted virus. Six independent experiments gave similar results and the data was presented as the mean of these values. Values are presented as infectivity ratio relative to K30 virus without expression of APOBEC3G.

We next examined whether APOBEC3G packaged into HTLV-1 virions deteriorated the infectivity of the virus. For this purpose, we employed the PCR-based infectivity assay as previously described [23] with modification because of very low infectivity of HTLV-1 virions. In brief, we prepared viruses from HEK293T cells transfected with K30 and expression vectors for APOBEC3G or its mutants and challenged these viruses to target SupT1 cells. Infectivity was determined by measuring HTLV-1 proviral DNA load in target cells with real-time quantitative polymerase chain reaction (RQ-PCR) [24]. To exclude the possibility that the residual viral DNA in the supernatant was detected by PCR method, we treated viruses with DNase before assay and prepared heat-inactivated virus as a negative control. Infectivity of K30 was suppressed almost to the level of that of heat-inactivated virus when expressed with APOBEC3G, its mutants, and muAPOBEC3G (Fig. 2 and data not shown). Interestingly, all the APOBEC3G inactive mutants also lowered the infectivity, suggesting that the enzymatic activity of APOBEC3G was dispensable for the antiviral activity on HTLV-1 and that APOBEC3G might act on HTLV-1 through different mechanisms.

### APOBEC3G did not induce G-to-A hypermutation in HTLV-1 genome

**Figure 3:**
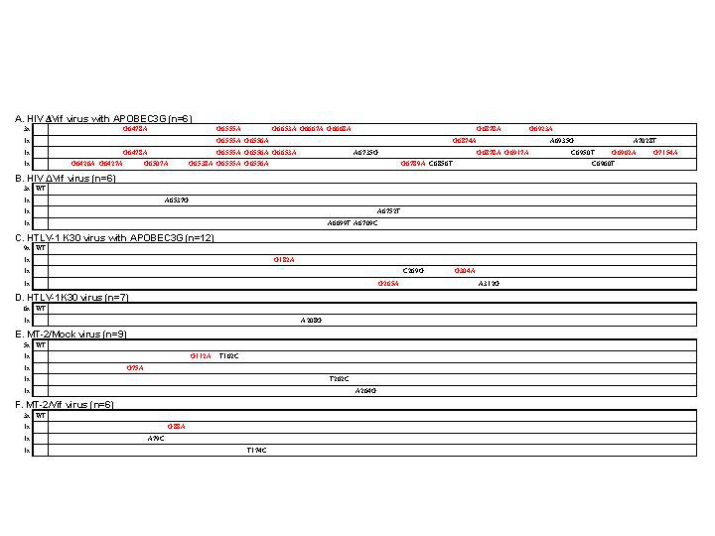
**No G-to-A hypermutation in HTLV-1 genome was induced by APOBEC3G**. Mutations in HTLV-1 and HIV-1 ΔVif viruses were detected by sequencing p12 and Env regions, respectively. G-to-A hypermutation was induced by APOBEC3G in HIV-1 ΔVif DNA, but not in HTLV-1 DNA. We detected very few G-to-A mutations in HTLV-1 K30 genome with expression of APOBEC3G (C), but not without expression of APOBEC3G (D), whereas G-to-A hypermutation was induced in HIV-1ΔVif DNA by APOBEC3G (A). We also detected a very few G-to-A mutations in MT-2/Mock virus DNA (E) as well as MT-2/Vif virus DNA (F). G-to-A mutations are shown in red, while other mutations are denoted in black. The numbers before the sequence indicate the number of each clone, while those in parentheses indicate the total number of clones sequenced. WT indicates no mutations in this region.

To confirm the above hypothesis, we examined whether APOBEC3G induces G-to-A hypermutation in HTLV-1 DNA. p12 region was amplified from target cell DNA and sequenced. We detected a few G-to-A mutations in HTLV-1 K30 genome integrated into target cell DNA in the presence of APOBEC3G (Fig. 3C), but not in the absence of APOBEC3G (Fig. 3D). These G-to-A mutations were only seen with expression of APOBEC3G and mostly occurred in the context of GpG sequence which is the preferred substrate for APOBEC3G, suggesting that these mutations were induced by APOBEC3G, although the frequency is very low as seen with HBV [14]. In contrast, G-to-A hypermutation was induced in HIV-1ΔVif DNA by APOBEC3G (Fig. 3A) as previously reported [3,6-8]. Accordingly, this again suggests the former notion that hypermutation may not be necessary for the antiviral activity of APOBEC3G on HTLV-1.

### HIV-1 Vif reverses the infectivity of HTLV-1 suppressed by endogenous APOBEC3G

**Figure 4:**
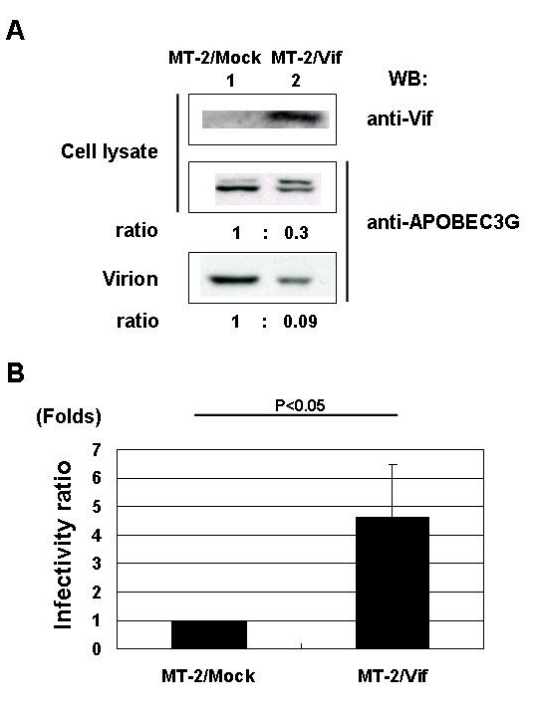
**HIV-1 Vif reduced the incorporation of APOBEC3G into HTLV-1 virions, resulting in the upregulation of the infectivity**. **(A) Expression of APOBEC3G in MT-2 cells and its incorporation into produced virions were reduced by HIV-1 Vif. **Expression level of APOBEC3G was reduced in MT-2/Vif cells (lane 2, middle panel) as compared to MT-2/Mock cells (lane 1, middle panel). Incorporation of APOBEC3G into produced virions was also reduced in virions produced from MT-2/Vif cells (lane 2, bottom panel). Expression of Vif protein in MT-2/Vif cells was detected with anti-Vif mAb (top panel). **(B) HIV Vif upregulated the infectivity of HTLV-1 produced from MT-2 cells. **Infectivity of HTLV-1 virus produced from MT-2 cells was determined as described in Materials and Methods. Infectivity of viruses produced from MT-2/Vif cells was more than 4 times higher than that from MT-2/Mock cells. Four independent experiments gave similar results and the data was presented as the mean of these values. Values are presented as infectivity ratio relative to viruses from MT-2/Mock cells.

Finally, we examined the antiviral activity of endogenous APOBEC3G. First, we confirmed the function of endogenous APOBEC3G in MT-2 cells by infection with HIV-1 wild type (WT) and ΔVif virions. WT virus could replicate in MT-2 cells, but ΔVif virus not (data not shown), indicating that endogenous APOBEC3G in MT-2 cells may be able to function as an anti-HIV-1 factor or that there may exist other APOBEC3 protein members sensitive to Vif. Based on this result, we performed an infectivity assay using HTLV-1 virions produced from MT-2 cells. Since we found that endogenous APOBEC3G was incorporated into HTLV-1 virions produced from MT-2 cells (Fig. 1D), we introduced HIV-1 Vif into MT-2 cells to see whether Vif can upregulate the infectivity of HTLV-1 virions produced from MT-2 cells by blocking the virion incorporation of APOBEC3G. MT-2/Mock and MT-2/Vif cell lines were established for this purpose using retrovirus vectors. We confirmed that Vif reduced expression of APOBEC3G in MT-2/Vif cells as well as its incorporation into produced virions (Fig. 4A). Unfortunately, expression of Vif was not enough to totally suppress the expression of APOBEC3G in MT-2/Vif cells and there were some levels of virion incorporation of APOBEC3G left. In order to affirm the inhibitory activity of HIV-1 Vif against APOBEC3G, we performed an infectivity assay using virions produced from these cell lines. The infectivity of viruses produced from MT-2/Vif cells was more than 4 times higher than that from MT-2/Mock cells (Fig. 4B). The infectivity assay on target cells after 10 days of culture also showed similar results (data not shown), suggesting that the possible detection of residual viral DNA in the culture was unlikely. These results indicate that endogenous APOBEC3G incorporated into HTLV-1 virions is functional and suppresses the infectivity of HTLV-1, which can be overcome by HIV-1 Vif. We also examined whether these proviruses have G-to-A hypermutation when integrated into the infected target cell DNA and again found very few G-to-A mutations in both viruses (Fig. 3E and 3F), suggesting that G-to-A hypermutation was not necessary for the inhibition of virus infectivity.

## Discussion

In this study, we have demonstrated that APOBEC3G has an antiviral activity on HTLV-1. APOBEC3G was efficiently incorporated into HTLV-1 virions and inhibited the infectivity of HTLV-1 without inducing G-to-A hypermutation. First, we showed that APOBEC3G, overexpressed or endogenous, was efficiently incorporated into HTLV-1 virions. Our finding suggests that HTLV-1 cannot exclude this protein from visions unlike HIV-1 [2-4,6-8]. Previous reports have shown that some accessory proteins encoded in open reading frames of HTLV-1 genome could enhance the infectivity of the virus. For example, deletion or mutants of p12 led to impaired infectivity of HTLV-1 both *in vivo *and *in vitro *[21,25]. We could not fully exclude the possibility that both K30 and the provirus in MT-2 cells possess mutations in some of these accessory genes so that these viruses could not exclude APOBEC3G from virions, although the possibility is quite low. Whether p12 potentially overcomes APOBEC3G has not been clarified and further investigations are necessary.

Second, we also showed that APOBEC3G inhibited the infection of HTLV-1. Because of low infectivity of cell-free HTLV-1 virions, we could not detect p19 production in the supernatant of infection culture (data not shown). Instead, we performed an infectivity assay as described previously with modification [23], in which RQ-PCR methods enabled us to quantify HTLV-1 genome integrated into target cells and measure the infectivity of cell free virions of HTLV-1, which was very low [24]. Using this method, we demonstrated that APOBEC3G suppressed the infectivity of HTLV-1. Interestingly, not only APOBEC3G but also its inactive mutants inhibited the infectivity of HTLV-1. Taken together with the data that APOBEC3G doesn't induce G-to-A hypermutation in HTLV-1 genome, these results indicate that the enzymatic activity is dispensable for the anti-HTLV-1 activity of APOBEC3G and that it may inhibit HTLV-1 through different mechanisms. In contrast, we previously reported that point mutants of C-terminal active site of APOBEC3G (E259Q, E67Q/E259Q) abrogated its antiviral activity on HIV-1, indicating that the enzymatic activity is essential for anti-HIV-1 activity of APOBEC3G [7]. Furthermore, some groups recently reported that APOBEC3G acts as an antiviral factor on HBV through several mechanisms [14,26]. One is induction of G-to-A mutations in cell type dependent manner, and the other is interference with pregenomic HBV RNA packaging without inducing G-to-A hypermutation. The reason why APOBEC3G inhibits HTLV-1 without inducing G-to-A hypermutation as seen with other retroviruses, even though it is a member of retroviruses, remains unclear. In order to elucidate the precise mechanisms of the antiviral activity of APOBEC3G on HTLV-1, further studies, such as its effects on translation of viral proteins, packaging of viral genome, and budding of virions, other than its cytidine deaminase activity, should be performed in the future.

To confirm the notion above, we prepared MT-2/Vif cells to block incorporation of endogenous APOBEC3G into HTLV-1 virions. Expression of Vif in MT-2 cells reduced the expression of APOBEC3G and its incorporation into virions. In the presence of Vif, APOBEC3G in MT-2 cells seemed to be ubiquitinated and degraded by the proteasome, because we detected two bands of APOBEC3G in MT-2/Vif cells by immunoblotting, of which the upper band might indicate mono-ubiquitinated APOBEC3G, while the faded lower band indicate the intact APOBEC3G remained (Fig. 4A, lanes 1 and 2, middle panel). Interestingly, we demonstrated that viruses released from MT-2/Vif cells recovered their infectivity which had been suppressed in MT-2/Mock cells. Then, we sequenced integrated HTLV-1 genome in target cells infected with viruses produced from MT-2/Vif and MT-2/Mock cells, and detected no G-to-A hypermutation (Fig. 3E and 3F). We hereby propose that the presence of functional endogenous APOBEC3G in virions from MT-2 cells inhibited the infectivity of the virus and that it might be linked to very low infectious titers of cell free HTLV-1 viruses. Taken together, our findings suggest that APOBEC3G might contribute to the unique features of HTLV-1 transmission, such as low infectivity of the virions [17] with very low genetic diversity [20].

During the preparation of this manuscript, Navarro et al. reported that HTLV-1 is relatively resistant to the antiviral effect of encapsidated APOBEC3G [27]. In that paper, they have shown that AOBEC3G is incorporated into HTLV-1 virion and suppresses the infectivity of HTLV-1, although the antiviral activity on HTLV-1 is very weak. We speculate that this discrepancy between their study and ours may originate from different assay systems to measure the infectivity of HTLV-1. They used a luciferase reporter HTLV-1 molecular clone in their study. However, luciferase activity was very low (below 10,000 cps) as compared to that of HIV-1 (more than 20 million cps). Taken together with our data that we could not detect the elevation of p19 levels in the supernatant of infection culture, we suspect that after integration the transcription level of viral gene is very low, resulting in low levels of luciferase activity and p19 production. In such a situation, luciferase reporter system might be inappropriate for evaluation of the infectivity of HTLV-1. Furthermore, in our study, we have shown that APOBEC3G inhibits HTLV-1 infection without exerting its cytidine deaminase activity, suggesting that APOBEC3G might act on HTLV-1 through different mechanisms from that on HIV-1. We believe that this is the first detailed report on the anti-HTLV-1 function of APOBEC3G and first description of possible involvement of other mechanisms than inducing G-to-A hypermutation in anti-HTLV-1 activity.

Finally, our findings have also broadened the spectrum of antiviral activity of APOBEC3G and further studies on the mechanisms of the antiviral activity of APOBEC3G on HTLV-1 will provide us with new insights into the function of this molecule as an antiviral innate immunity.

## Conclusion

APOBEC3G is incorporated into HTLV-1 virions and inhibits the infection of HTLV-1 without exerting its cytidine deaminase activity. This suggests that APOBEC3G might act on HTLV-1 through different mechanisms from that on HIV-1 and contribute to the unique features of HTLV-1 infection and transmission.

## Materials and methods

### Expression vectors and molecular clones

Expression vectors for hemagglutinin (HA)-tagged human APOBEC3G (APOBEC3G), its point mutants (E67Q, E259Q, and E67Q/E259Q), and murine APOBEC3G (muAPOBEC3G) were described previously [4,7]. pNL43-Luc and pNL43/Δvif-Luc were also constructed as previously described [7]. HTLV-1 K30 was a kind gift from Dr. Thomas Kindt through the AIDS Research and Reference Reagent Program [28]. The vif gene was amplified by PCR method from pNL43 and cloned into pDON-AI (Takara Bio Inc., Otsu, Japan) to construct a retrovirus vector, pDON/Vif.

### Cell lines

HEK293T cells were maintained in Dulbecco's modified Eagle's medium (Invitrogen, Carlsbad, California) containing 10% fetal calf serum, penicillin, streptomycin, and glutamine (Invitrogen). SupT1 cells and MT-2 cells were maintained in RPMI 1640 (Sigma, St. Louis, Missouri) containing 10% fetal calf serum, penicillin, streptomycin, and glutamine. MT-2/Mock and MT-2/Vif cells were established by transduction of retrovirus vectors (pDON-AI and pDON/Vif, respectively) and selection with Neomycin (Nacalai tesque, Kyoto, Japan).

### Expression of APOBEC3G in producer cells and its incorporation into visions

Western blotting was performed to detect expression of APOBEC3G, its mutants, and muAPOBEC3G in producer cells, and their incorporation into virions as described previously [4]. In brief, expression vectors for HA-APOBEC3G, its mutants, or HA-muAPOBEC3G were cotransfected with K30, pNL43-Luc, or pNL43/Δvif-Luc into HEK293T cells. Two days after transfection, viruses in the supernatant were collected and ultracentrifuged with Beckman TL-100s ultracentrifuge at 60,000 × g for 10min and subjected to sodium dodecyl sulfate-polyacrylamide gel electrophoresis (SDS-PAGE) together with whole cell lysates of producer HEK293T cells. To detect HA-tagged proteins, they were immunoblotted with anti-HA monoclonal antibody (mAb) (12CA5) (F. Hoffmann-La Roche Ltd., Basel, Switzerland). Virus production was confirmed by immunoblotting with the following antibodies; GIN-7(anti-p19 mAb)[29] for HTLV-1 and anti-p24 mAb (ZeptoMetrix Corporation, Buffalo, New York) for HIV-1. To detect endogenous APOBEC3G in MT-2 cells and its incorporation into virions, whole cell lysates of MT-2 cells and precipitated virions were subjected to immunoblotting with anti-APOBEC3G antibody (a kind gift from Dr. Warner C. Greene, Gladstone Institute of Virology and Immunology, University of California, San Francisco). Vif expression in MT-2/Vif cells was detected with anti-Vif mAb (#319) (a kind gift from Dr. Michael H. Malim through the AIDS Research and Reference Reagent Program) (18). Cytoplasmic proteins were detected with anti-β-tubulin mAb (D-10)(Santa Cruz Biotechnology, Santa Cruz, California). Samples applied to Western blotting were equalized according to p19 antigen levels for HTLV-1 and p24 antigen levels for HIV-1.

### Purification of HTLV-1 virions by sucrose density equilibrium gradients and analysis of APOBEC3G packaging

To confirm the incorporation of APOBEC3G into virion, HTLV-1 K30 virions were purified by sucrose density equilibrium gradients as previously reported with slight modifications [30]. Briefly, HTLV-1 K30 virions were prepared as described above and pelleted by ultracentrifugation, then resuspended in 150μl of PBS. They were laid on top of the sucrose gradient, prepared in PBS ranging from 10 to 60%, and centrifuged for 13 h at 20,000 rpm in an SW-41Ti rotor (Beckman, Palo Alto, California). Gradient fractions were collected from the top of the gradient. These samples were used for analyzing protein profiles of the virion by Western blotting. They were subjected to immunoblotting with anti-HA mAb (12CA5) and GIN-7 for detection of HA-APOBEC3G and p19, respectively.

### Assessment of HTLV-1 infectivity

Infectivity of HTLV-1 was detected as previously reported with slight modifications [23]. In brief, expression vectors for HA-APOBEC3G, its mutants, or HA-muAPOBEC3G were cotransfected with K30 into HEK293T cells. Viruses in the supernatants were collected 2 days after transfection, then treated with DNase (80 U/ml) (Roche Diagnostics GmbH, Germany) at 37°C for 1 h and filtrated through a 0.45-μm-pore-size filter. Viruses from MT-2 cells were also collected and treated in the same way. We also used noninfectious HTLV-1 as a negative control that had been heat inactivated at 56°C for 1 h. Virus titers were measured with an enzyme-linked immunosorbent assay kit for the p19 antigen (RETRO-TEK, ZeptoMetrix Corporation). SupT1 cells were challenged with viruses whose amounts were equalized according to p19 antigen levels, and washed five times after incubation at 37°C for 8 h. These target cells were cultivated for 2 to 10 days and total cellular DNA was extracted with DNA Mini kit (Quiagen, Valencia, California). HTLV-1 proviral DNA loads were measured by RQ-PCR as described previously [24].

### Detection of mutations in the viral DNA

Mutations in HTLV-1 DNA were detected by sequencing p12 region of HTLV-1 integrated into target cells [4]. Preparation of total cellular DNA of target cells infected with HTLV-1 is described above [23]. The p12 region of HTLV-1 was amplified with the following primer pairs:op-32.1(ATAGTCGACCTGTTTCGCCTTCTCAGCCC) and op-32.3(TATCTCGAGGAAGCTGTGCTTGACGG). The PCR products were cloned into pT7-Blue (Novagen, Darmstadt, Germany) and the inserts of individual clones were sequenced. Mutations in HIV-1 NL43 Env region were also detected as previously described [7].

## Competing interests

The author(s) declare that they have no competing interests.

## Authors' contributions

AS designed research, performed research, contributed vital new reagents, analyzed data, and wrote the paper. AT-K designed research, performed research, contributed vital new reagents, analyzed data, wrote the paper, and organized research. KS performed a part of research. MK performed a part of research. AA performed a part of research. MH performed a part of research and contributed vital new analytical tools. KI contributed vital new analytical tools and analyzed data. YT contributed vital new reagents. TU analyzed data, drafted the paper, and organized research.
